# Annotations

**Published:** 1897-06-19

**Authors:** 


### ANNOTATIONS.

### The Health of the Queen.

We have much pleasure in being able to deny, on the best authority, certain disquieting paragraphs which have recently appeared in the press in reference to the Queen and the Royal Family. It has been stated that Her Majesty has felt much anxiety in regard to the procession, and has been troubled by anxious forebodings of accident to herself or her subjects. Various statements have also appeared as to the mental health of "one of the prettiest of the Royal Princesses." We are assured that all these rumours are absolutely without foundation and contrary to fact, and it has been pointed out to us that, so far from the Queen dreading her public appearance, the Kensington ceremonial is an additional labour voluntarily undertaken. We must remember that at the Queen's age the ordeal is likely to be a trying one, but we can absolutely contradict the statement that it is one in regard to which Her Majesty entertains either any dread or any foreboding of disaster.

### Disinfection of Infected Rooms.

The discussion which has recently taken place at the Society of Medical Officers of Health as to the best means of disinfecting rooms after they have been occupied by patients suffering from infectious disorders cannot be said to have ended in any very definite pronouncement. The principal methods discussed were those by sulphurous acid, by formic aldehyde, by washing with disinfectant solutions, or with mere soap and water, and by spraying with solution of perchloride of mercury. The difficulty of obtaining anything like certainty of disinfection by means of these agents is greater than many people imagine. If sulphurous acid is to be produced in anything like bactericidal proportions much more sulphur must be used than is ordinarily done; while in spraying, the completeness with which the process is carried out depends entirely upon the honesty and conscientiousness of the workmen employed, and the labour involved is very considerable. The vapour of formic aldehyde seems to have comparatively little effect upon fabrics, and it has been well reported on by many observers, but more experience as to its efficiency is desirable; on the other hand, washing with disinfectants, although effective enough, is destructive to many papers and other decorative materials. The question naturally arises whether the fact of a method of disinfection being destructive to decorative objects should be allowed to stand in the way of its use. Difficulties only arise where the rooms are in good condition and it is desired not to remove the paper, and in regard to this we cannot help feeling that in proportion to his power of bearing the loss the owner of a mansion will suffer less from having even a fine paper stripped from the walls than the dweller in a small tenement often does from the disinfection which is forced upon him by the sanitary authorities. The only non-destructive process which can practically be said to be "in the market" is that by formic aldehyde, and as to the value of this some authoritative pronouncement is urgently required. The decision of this question would seem to come eminently within the functions of the medical department of the Local Government Board.

